# Determinants of culinary Students’ Entrepreneurial Intentions: The role of social media use within dual-theory frameworks

**DOI:** 10.1371/journal.pone.0342055

**Published:** 2026-02-05

**Authors:** Xinling Zhong

**Affiliations:** School of Culinary and Food Science Engineering, Sichuan Tourism University, Chengdu, Sichuan, China; Pingdingshan University, CHINA

## Abstract

China’s national “mass entrepreneurship and innovation” strategy has sparked growing attention toward cultivating entrepreneurial competencies among university students. As a vital resource and interactive platform, social media profoundly shapes the entire entrepreneurial process. However, existing research lacks a systematic examination of the mechanisms through which social media influences entrepreneurial intentions (EI)—particularly among culinary students. Grounded in Social Cognitive Theory (SCT) and the Theory of Planned Behavior (TPB), this study examines the influence of social media use (SMU) on EI among culinary students. Using a snowball sampling method, the researchers surveyed 499 students, collecting self-reported responses across six constructs: SUM, subjective norms (SN), entrepreneurial self-efficacy (ESE), entrepreneurial attitude (EA), perceived behavioral control (PBC), and EI. A two-stage analytical approach combining Structural Equation Modeling and Artificial Neural Networks was employed to uncover the constructs’ complex, nonlinear, and non-compensatory relationships. The results revealed that SMU does not directly influence EI; instead, it exerts a positive indirect effect through the mediating roles of PBC, ESE, and EA. Moreover, the normalized importance derived from the multilayer perceptron indicated that EA (100%) is the most significant predictor of entrepreneurial intention among culinary arts students. This study offers theoretical and practical insights into how social media can be leveraged to foster entrepreneurship among students in the culinary field.

## Introduction

Entrepreneurship is widely regarded as a key driver of national economic growth, playing a vital role in promoting employment, alleviating unemployment, stimulating innovation, and enhancing labor market competitiveness [1]. University students at a critical stage of career planning and transition represent an important pool of potential future entrepreneurs [2]. By 2025, China’s strategy of “mass entrepreneurship and innovation” has evolved into an integrated “fourfold innovation linkage” ecosystem, aimed at improving the quality of student employment and entrepreneurship while supporting their sustainable career development [3,4]. Nevertheless, despite the growing policy support, the entrepreneurial intention (EI) of Chinese university students remains as low as 5.1%, ranking second to last among 57 countries [5].

In the digital era, social media—functioning as an integrated digital platform for social interaction, knowledge sharing, and marketing communication—has become increasingly embedded in the daily lives of university students. Prior research has demonstrated that social media can shape individual cognition, social identity, and behavioral intentions [6]. Existing literature has primarily focused on the impact of social media on students’ social interactions [7,8] and academic performance [9,10], highlighting its potential role in shaping cognition and behavior. However, systematic investigations still lack how social media affects students’ entrepreneurial cognition, self-efficacy, perceived behavioral control (PBC), and EI. However, how social media use (SMU) influences entrepreneurial intention (EI), particularly through entrepreneurial cognition such as self-efficacy and perceived behavioral control (PBC), remains insufficiently explored.

In previous studies of EI, Social Cognitive Theory (SCT) and the Theory of Planned Behavior (TPB) are two widely adopted theoretical frameworks [1,11–13]. The SCT emphasizes the cognitive regulatory mechanisms that arise from the interaction between individuals and their environment. It is particularly suitable for explaining internal variables such as personal capability, self-efficacy, and goal-directed behavior. Conversely, the TPB explains behavioral intention formation by highlighting the roles of subjective norms (SN), attitudes, and PBC. Each theory captures important but partial mechanisms of entrepreneurial intention, suggesting that their integration is particularly suitable for examining social media–embedded entrepreneurial cognition. Therefore, integrating SCT and TPB offers a complementary perspective, capturing both cognitive and social influences, thereby constructing a more comprehensive mechanism for understanding the formation of EI.

Methodologically, Structural Equation Modeling (SEM) has been widely applied to model and test relationships among entrepreneurship-related variables. For instance, Niu, Niu [14] found that social support and creativity indirectly influenced students’ EI through entrepreneurial self-efficacy (ESE) and entrepreneurial attitude (EA). However, SEM primarily captures linear relationships, which limits its ability to model complex nonlinear patterns. Recent studies have increasingly incorporated Artificial Neural Networks (ANN) to capture complex nonlinear structures and rank variable importance to address this limitation, enhancing the model’s predictive accuracy and explanatory power.

Although numerous studies have examined the factors influencing university students’ EI, most have focused on traditional disciplines with stronger entrepreneurial tendencies, such as business and engineering [15]. In contrast, there has been an apparent lack of exploration into students’ entrepreneurial cognition and behavioral pathways in non-traditional fields, such as culinary arts. Moreover, prior research has largely emphasized formal institutional factors, while paying limited attention to the influence of high-frequency informal behaviors, such as SMU, on entrepreneurial intention.

Accordingly, this study aims to integrate SCT and the TPB and, by employing a combined SEM and ANN approach, to examine how SMU influences the EI of culinary arts university students through PBC, SN, EA, and ESE. To this end, the study addresses the following research questions:

1. Does SMU significantly influence the EI of culinary arts university students?
2. Does SMU indirectly affect EI through psychological constructs?
3. Based on ANN, which constructs demonstrate the strongest predictive power for EI?

The potential contributions of this study lie in four main areas. First, examining SMU fills a theoretical gap in entrepreneurship research within non-traditional disciplines. Second, integrating the TPB and SCT frameworks proposes a more systematic cognitive model of entrepreneurship, extending the theoretical boundaries of research on EI. Third, adopting a hybrid SEM–ANN approach enhances the explanatory and predictive power of relationships among constructs. Finally, the study uncovers several path relationships that diverge from prior findings, thereby identifying boundary conditions for the applicability of existing theories and offering new directions for future research.

The structure of this paper is arranged as follows. Section 2 presents the literature review. Section 3 introduces the research hypotheses and theoretical model. Section 4 describes the research methodology. Section 5 reports the data analysis and empirical results. Section 6 discusses the research findings, theoretical and practical implications, limitations, and directions for future research. Finally, Section 7 provides the conclusion.

## Literature review

### Social cognitive theory

SCT, first proposed by Bandura [16], posits that human activities are shaped by a triadic reciprocal interaction among personal, behavioral, and environmental factors, emphasizing the critical role of learning experiences in developing individual behavior [17]. The theory argues that individuals acquire new cognitions and perceptions of their environment through observational learning during specific actions, influencing their future behaviors [18]. The SCT encompasses three key dimensions: personal, behavioral, and environmental factors [16,19]. Rather than operating in isolation, personal cognition, behavior, and environmental conditions dynamically interact and mutually influence one another, forming a reciprocal framework for understanding behavior formation.

SCT has been widely applied across diverse fields and has yielded significant insights into various forms of human behavior. In academic entrepreneurship, Liao, Nguyen [20] found that scholars strengthen their ESE through continually transforming research outputs, while observational learning—such as examining peers’ entrepreneurial cases and engaging in technology transfer activities—stimulates EI. In artificial intelligence entrepreneurship, Sutrisno, Siminto [21] reported that ChatGPT directly enhanced students’ EI and indirectly reinforced them by stimulating innovative behavior. This study focuses on the relationship between SMU and EI among culinary arts students, a context in which SCT is particularly well-suited. On one hand, students’ self-efficacy and entrepreneurial motivation are often strengthened through the display of their work on social media and the feedback they receive from others, exemplifying the role of *personal factors* within SCT. On the other hand, by observing others’ entrepreneurial experiences and participating in online interactions, students engage in learning and imitation processes that reflect *behavioral factors*. Furthermore, social media functions as an essential environmental resource platform for entrepreneurship, illustrating the role of *environmental factors*. Taken together, SCT’s triadic reciprocal model provides a systematic framework for explaining the formation of EI among culinary arts students in SMU.

Moreover, as academic research has advanced, SCT has increasingly been integrated with other theories to enhance its explanatory and predictive power. For example, in entrepreneurship education research, Liu [22] combined Goal Orientation Theory, Social Capital Theory, and SCT to construct an integrated model for explaining the formation of students’ EI. The findings confirmed that the integrated theoretical model exhibited greater explanatory capacity than single-theory approaches. Similarly, Nguyen and Nguyen [23] combined SCT with the TPB to uncover students’ entrepreneurial passion drivers, significantly improving the explanatory power for emotion-driven entrepreneurial behaviors. These findings provide an important theoretical basis for adopting an integrated SCT–TPB framework in the present study.

Despite the expanding application of SCT and its significant contributions across various fields, research on entrepreneurial behavior still exhibits notable gaps. First, most studies have concentrated on business or engineering students. At the same time, insufficient theoretical attention has been given to students’ entrepreneurial mechanisms and cognitive processes in non-traditional disciplines such as culinary arts [15]. Second, existing literature has primarily focused on offline environmental factors—such as family background and school education—while relatively few studies have examined the relationship between digital media environments and entrepreneurial behavior [24]. Given the ubiquity of SMU among students and the distinctive cognitive characteristics of culinary arts students, investigating the link between SMU and EI in this group offers an important opportunity to enrich SCT’s research perspective. This study seeks to fill this theoretical gap by analyzing the specific mechanisms through which SMU shapes EI, thereby providing both new theoretical contributions and empirical evidence for the advancement and practical application of SCT.

### Theory of planned behavior

The TPB, first proposed by Ajzen [25], is primarily employed to explain why individuals form specific behavioral intentions and how these intentions translate into actual behaviors. The theory posits that behavioral intention is jointly determined by an individual’s attitude toward the behavior, SN, and PBC, which in turn influence the occurrence of the behavior [26]. As a result, TPB provides a parsimonious and widely validated framework for explaining the motivational structure underlying entrepreneurial intention [27].

In recent years, the TPB has been widely applied across multiple research domains and has achieved notable success in predicting and explaining human behavior. In sustainable entrepreneurship, Yasir, Babar [28] found that a positive attitude toward sustainable entrepreneurship directly strengthened EI, while supportive SN indirectly reinforced intention by enhancing perceived social support. In the entrepreneurship education, Slomski, Tavares de Souza Junior [29] demonstrated that PBC not only directly enhanced EI but also indirectly strengthened it by fostering a positive attitude. In the digital entrepreneurship, Garcez, Franco [30] revealed that favorable EA and supportive SN both directly promoted EI and indirectly enhanced it through improved PBC. These findings suggest that TPB provides a robust framework for examining how cognitive evaluations and perceived control shape entrepreneurial intention. In the present study, TPB is particularly suitable for analyzing the entrepreneurial intention of culinary arts students in a social media context, where attitudes, perceived social expectations, and control perceptions are likely to be shaped by digitally mediated experiences.

In addition, as academic research has progressed, the TPB has increasingly been integrated with other theories to enhance its explanatory and predictive power. For example, Ferri, Spanò [31] expanded the explanatory scope of the TPB in general higher education by incorporating the skill dimension, thereby deepening the understanding of students’ EI formation. However, the limited consideration of subjective traits and contextual environmental factors restricts the generalizability of this model and its explanatory strength across diverse entrepreneurial domains. In entrepreneurship education research, Maheshwari and Kha [32] combined the TPB with self-efficacy theory to examine the psychological and behavioral drivers of university students’ EI. This theoretical integration significantly improved the accuracy of predicting EI. Nonetheless, the model remains underdeveloped in incorporating broader macro-environmental contexts, such as policy frameworks and cultural settings, which must be strengthened to enhance its explanatory power across different contexts of entrepreneurship.

Despite the expanding applications and significant advancements of the TPB, existing research still reveals notable limitations. Most studies have focused on the influence of entrepreneurship education in higher education on students’ EI. At the same time, relatively little theoretical attention has been paid to new contextual factors such as SMU [32]. Given the pervasiveness of social media, examining the relationship between SMU and EI among culinary arts students offers an important opportunity to enrich the TPB research perspective. This study seeks to fill this theoretical gap by analyzing how culinary students’ attitudes, SN, and PBC evolve within SMU, thereby uncovering how these factors influence EI. In doing so, it provides new theoretical contributions and empirical evidence for the further development and practical application of the TPB.

### Theoretical integration of social cognitive theory and the theory of planned behavior

Although social cognitive theory (SCT) and the theory of planned behavior (TPB) have each been extensively applied to explain entrepreneurial intention, their explanatory focus differs in important ways. TPB primarily addresses the motivational and intentional structure of entrepreneurship by clarifying how attitudes, subjective norms, and perceived behavioral control jointly shape individuals’ entrepreneurial intention. However, TPB provides limited insight into how these cognitive evaluations are formed through learning experiences and capability development, particularly in educational contexts.

In contrast, SCT emphasizes how entrepreneurial cognition is constructed, highlighting the roles of observational learning, experiential engagement, and self-efficacy in shaping individuals’ beliefs about their entrepreneurial competence. SCT is therefore well suited to explain how students’ entrepreneurial abilities and confidence are cultivated through social media–supported interaction and feedback. Nevertheless, SCT alone does not explicitly model how such capability-related cognitions are translated into deliberate entrepreneurial intention.

By integrating SCT and TPB, the present study captures both capability formation and intention formation within a unified framework. SCT explains how social media use contributes to the development of entrepreneurial self-efficacy and abilities, while TPB clarifies how these capability-based cognitions, together with attitudes, subjective norms, and perceived behavioral control, shape entrepreneurial intention. This integrative perspective provides superior explanatory power by linking learning-driven cognitive development with intentional decision-making, thereby offering a more comprehensive explanation of entrepreneurial intention among culinary arts students.

### Entrepreneurial intention

EI refers to an individual’s psychological tendency and determination to start a business or engage in entrepreneurial activities, and it is widely recognized as a critical antecedent of entrepreneurial behavior [33]. Research on EI has drawn upon multiple theoretical frameworks, including the TPB [1,13,33], SCT [11,34], Social Capital Theory [22], and the Conservation of Resources Theory [5]. The TPB and SCT are the most widely applied and relevant to the present study. The TPB, as a foundational framework for explaining the formation of EI, posits that EI is determined by an individual’s attitude toward entrepreneurial behavior, SN, and PBC. For example, drawing on the TPB, Boucif, Wan Nawang [1] found that attitude directly predicts EI and indirectly influences it by shaping PBC. However, the TPB primarily emphasizes psychological cognition while paying insufficient attention to external environmental dynamics—such as policy, social resources, and cultural context—thus limiting its capacity to explain the mechanisms underlying EI fully. By contrast, SCT suggests that EI emerges from the reciprocal interaction of personal factors, environmental influences, and behavioral outcomes. For instance, Nwosu, Obidike [34], building on SCT, investigated how environmental factors (e.g., internships in commercial firms) and personal factors (e.g., entrepreneurial self-efficacy, creativity, and passion) shape students’ EI. Their findings showed that internships indirectly fostered EI by enhancing students’ ESE and creativity. Nevertheless, SCT emphasizes internal cognitive processes and direct interactions with the environment, while giving comparatively less attention to social influences such as group pressure and SN. As a result, it struggles to explain how these social factors drive cognitive convergence among individuals.

Scholars have increasingly employed quantitative methods to examine the factors influencing students’ EI in recent years. Common quantitative approaches include correlation analysis [35], regression analysis [11,36], mediation analysis [37], and SEM [38,39]. For example, Ghouse, Barber Iii [11] used regression analysis to investigate the formation mechanisms of rural students’ EI. They found that self-efficacy directly enhanced individuals’ confidence in their entrepreneurial abilities, strengthening EI. Through large-scale data collection and statistical analysis, quantitative research allows for precise measurement and examination of the strength of EI, its influencing factors, and the interrelationships among them. However, such research often places heavy emphasis on numerical analysis while overlooking the complex social, psychological, and cultural factors underlying EI, which may limit its ability to capture the multidimensional nature of the phenomenon entirely.

Studies have shown that personal, environmental, and sociocultural factors are important antecedent predictors of EI. First, *personal factors* include personality traits [40], self-efficacy [41], motivation [42], gender [43], and attitudes [44]. For instance, Schlaegel, Engle [40] examined the influence of personal factors—including personality traits and abilities—on EI. They found that innovative personality traits indirectly enhanced entrepreneurial intention by increasing the likelihood of identifying entrepreneurial opportunities. However, personal factors alone cannot account for the broader macro-environmental and sociocultural contexts in which EI are formed. Second, *environmental factors* include government policies [45], the economic environment [46,47], entrepreneurship education [29], and institutional settings [48]. For example, based on the theory of entrepreneurial intention, Požega and Ribić [46] investigated the impact of the economic environment on EI. They found that regional economic development levels influenced students’ perceptions of entrepreneurial opportunities and risks, shaping their EI. Nevertheless, environmental factors cannot fully explain internal motivations and individual choices; even in regions with favorable economic conditions and strong policy support, not all individuals pursue entrepreneurship. Third, *sociocultural factors* include social support [49], SN [50], social values [51], and social networks [52]. For instance, Neneh [49] examined the influence of entrepreneurial passion, ESE, and social support on students’ EI and found that social support directly enhanced confidence and motivation toward entrepreneurship, thereby strengthening EI. At the same time, social support indirectly promoted EI by reinforcing the effect of entrepreneurial passion on self-efficacy. However, while sociocultural factors shape individuals’ perceptions, attitudes, and values toward entrepreneurship, they cannot fully predict entrepreneurial behavior. Even in supportive sociocultural environments, not all individuals are willing to bear the risks and pressures associated with entrepreneurship.

## Hypotheses

### Social media use, subjective norms, and entrepreneurial intention

Social media is an effective tool for connecting individuals, gathering additional information, and analyzing knowledge [53]. While prior studies have operationalized social media use in terms of frequency, duration, and intensity [54], the present study conceptualizes social media use (SMU) as entrepreneurial-oriented engagement with social media platforms, focusing on acquiring restaurant entrepreneurship knowledge, observing entrepreneurial practices, and interacting with relevant professional communities, rather than general recreational use.

SMU refers to students’ engagement with social media platforms, specifically in frequency, duration, and intensity [54]. Several studies have demonstrated a significant association between SMU and EI [55,56]. For example, drawing on the TPB, Loan, Libo-on [55] found that students who used social media to access entrepreneurial information and knowledge while building broader networks for support and resources exhibited stronger EI. Similarly, Al Halbusi, Soto-Acosta [56], examining the determinants of e-entrepreneurial intention among Iraqi university students, revealed that exposure to peers’ or entrepreneurs’ social media content fostered more positive affective evaluations of e-entrepreneurship, thereby enhancing EI. In line with these findings, this study proposes the following hypothesis:

**H1:** SMU has a significant positive effect on the EI of culinary arts students.

SN refers to the expectations and social pressure perceived from significant others or social groups when an individual makes decisions [57]. Prior studies have demonstrated that SMU significantly influences students’ SN [55,58]. For example, drawing on the TPB, Loan, Libo-on [55] found that SMU enhanced students’ SN by expanding their social networks and increasing interactions on digital platforms. Similarly, Barrera-Verdugo and Villarroel-Villarroel [58] examined the relationship between SMU frequency and the EA of Chilean business and engineering students, revealing that social media, as an external environmental factor, amplified students’ perceptions of social pressure through information dissemination and social interaction, thereby strengthening their SN. In line with these findings, this study proposes the following hypothesis:

**H2:** SMU has a significant positive effect on the SN of culinary arts students.

Numerous studies have demonstrated that SN significantly positively influence EI [32,59]. For instance, drawing on the TPB, Maheshwari and Kha [32] examined how entrepreneurship education support shaped the EI of Vietnamese university students and found that SN fostered EI by enhancing students’ entrepreneurial confidence. Similarly, Duan [59] investigated the factors influencing Chinese university students’ EI and reported that SN strengthened EI by increasing students’ confidence and motivation while reducing concerns about entrepreneurial risks. Accordingly, this study proposes the following hypothesis:

**H3:** SN significantly positively affect the EI of culinary arts students.

**H4:** SN mediate the relationship between SMU and EI among culinary arts students.

### Social media use, entrepreneurial self-efficacy, entrepreneurial attitude, and entrepreneurial intention

ESE refers to an individual’s belief in their capability to perform tasks and achieve outcomes associated with entrepreneurship [60]. Prior studies have demonstrated that SMU significantly affects SES [61,62]. For instance, Kurnia Khafidhatur, Irsyad [61] showed that positive interactions on social media can enhance students’ ESE. Similarly, drawing on SCT and the TPB, Hu, Jabor [62] found that SMU, by providing access to skill-learning resources and peer interaction support, strengthened vocational students’ confidence in their entrepreneurial abilities, thereby improving their ESE. Based on these findings, this study proposes the following hypothesis:

**H5:** SMU has a significant positive effect on the ESE of culinary arts students.

EA refers to an individual’s overall positive or negative evaluation of specific entrepreneurial behaviors or actions [63]. It is not merely an expression of intention but also reflects an inherent disposition toward entrepreneurship as a particular goal [64]. Prior research has established a significant relationship between ESE and EA [65–67]. For example, within an integrated framework of the TPB and SCT, Cao [65] found that higher ESE strengthens individuals’ confidence in their entrepreneurial capabilities, fostering more positive EA. Similarly, drawing on the Cognitive-Affective Processing Systems Theory, Song and Lu [67] demonstrated that ESE shapes EA by boosting confidence, reducing fear of failure, enhancing expectations of success, and improving adaptability. Accordingly, this study proposes the following hypothesis:

**H6:** ESE has a significant positive effect on the EA of culinary arts students.

Multiple studies have shown that students’ EA significantly and positively influence their EI [68,69]. For example, drawing on the TPB, Lim, Kim [68] found that positive EA strengthened nursing students’ confidence in their abilities and willingness to take risks, enhancing EI. Similarly, Qudsia Yousaf, Munawar [69], examining the effects of entrepreneurship education, EA, and culture on Pakistani students’ EI, reported that students with more positive attitudes toward entrepreneurship were more inclined to identify and evaluate entrepreneurial opportunities. Moreover, positive affect fostered by EA stimulated intrinsic motivation, reinforcing students’ determination to overcome obstacles and embrace challenges, strengthening EI. Accordingly, this study proposes the following hypothesis:

**H7:** EA has a significant positive effect on the EI of culinary arts students.

**H8:** ESE and EA mediate the relationship between SMU and EI among culinary arts students.

### Social media use, perceived behavioral control, and entrepreneurial intention

PBC refers to the perceived ease or difficulty of performing a specific behavior [25], reflecting an individual’s evaluation of their ability to carry out entrepreneurial activities [70]. Numerous studies have shown that SMU significantly influences PBC [55,71,72]. For example, Huang and Zhang [71] demonstrated that SMU provides students with entrepreneurial knowledge and information, directly enhancing their ESE. In turn, students with higher self-efficacy feel more confident in overcoming challenges and difficulties, improving their PBC [72]. Similarly, Loan, Libo-on [55] found that SMU enhanced Vietnamese students’ PBC through multiple mechanisms, such as providing information, building relationships, sharing experiences, and offering convenience, collectively boosting their confidence in entrepreneurial capabilities. Based on these findings, this study proposes the following hypothesis:

**H9:** SMU has a significant positive effect on the PBC of culinary arts students.

Prior studies have confirmed a significant relationship between PBC and students’ EI [73,74]. For example, drawing on the TPB, Lim, Kim [73] examined the factors influencing nursing students’ EI. They found that students with higher PBC were more likely to enhance their skills and knowledge through entrepreneurship education, strengthening their ESE and ultimately their EI. Similarly, Sargani, Jiang [74] investigated gender differences in the EI of Pakistani agricultural students. They revealed that students with higher PBC were more inclined to seek and accept support from family, friends, and society. This reinforced their positive attitudes toward entrepreneurship and, in turn, increased their EI. Based on these findings, this study proposes the following hypothesis:

**H10:** PBC has a significant positive effect on the EI of culinary arts students.

**H11:** PBC mediates the relationship between SMU and EI among culinary arts students.

**Fig 1:**
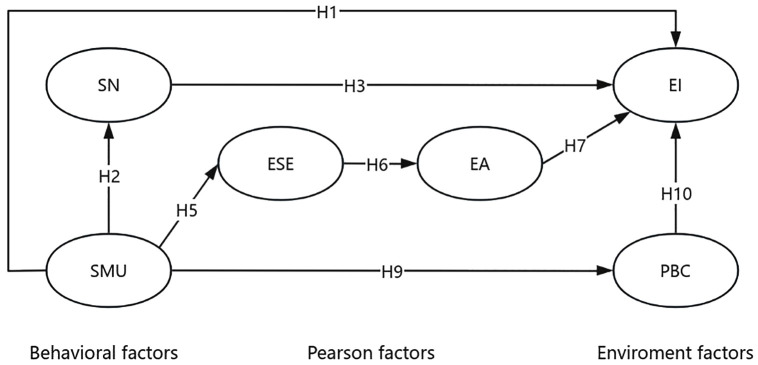
Research model.

Based on the above literature review and hypotheses, the hypothetical model of this study is shown in Fig 1.

## Methodology

### Participants

Before completing the questionnaire, participants were provided with detailed information about the study, including its purpose and the protection of participant privacy. Only those who gave written informed consent were allowed to proceed, and participants retained the right to withdraw at any time. Data was collected from March 10 to April 25, 2025, via the Wenjuanxing platform (https://www.wjx.cn/). This platform was chosen because of its professional data collection functions, strong adaptability for Chinese users, and robust privacy safeguards, making it particularly suitable for large-scale and remote data collection while ensuring research rigor and data quality. A snowball sampling method was employed, whereby initial participants referred new participants who met the eligibility criteria, creating a chain-referral network that gradually expanded the sample size while maintaining sample relevance. Participants were also encouraged to recommend other suitable respondents to join the survey. As a non-probability sampling technique, snowball sampling may introduce selection bias by over-representing individuals with stronger social connections or greater interest in entrepreneurial activities; therefore, the findings should be interpreted with appropriate caution regarding their generalizability. In addition, Wenjuanxing’s response-time recording function was used to monitor and assess data quality. Respondents who completed the survey and provided valid answers were given a random cash reward.

This study collected data from 558 undergraduate students at three universities in Sichuan Province, China. To ensure sample quality, we followed the procedure of Wu, Li [75] and applied three screening criteria. First, based on preliminary testing, the average completion time for the questionnaire ranged from two to five minutes. Participants who completed the survey in less than 90 seconds were considered to have responded carelessly, and their data were excluded. Second, the questionnaire contained one reverse-coded item; participants who failed to provide the correct reverse-coded response were deemed inattentive, and their data were removed. Third, questionnaires with identical responses across all items were also excluded. After this rigorous screening process, 59 invalid responses were discarded, leaving 499 valid questionnaires for further analysis. In the hypothesized model of this study, the maximum number of arrows pointing to an endogenous latent construct was four. According to Hair [76], a minimum sample size of 191 is required to achieve an R² value of 0.10 at the 1% significance level. The final sample size of 499 participants far exceeded this threshold, ensuring the robustness and reliability of the study’s findings.

Among the 499 valid questionnaires, 170 were male (34.1%) and 329 were female (65.9%). In terms of grade distribution, 227 were freshmen (45.5%), 134 were sophomores (26.9%), 122 were juniors (24.4%), and 16 were seniors (3.2%) (Table 1). This representative sample provides a solid foundation for analyzing SMU’s impact on culinary arts students’ EI. The sample’s diversity and size help ensure that this study’s findings broadly apply to the broader population of university students within this research context.

**Table 1:** Demographic information of participants (N = 499).

Demographic information	Category	Frequency	Percentage(%)
Gender	Male	170	34.1
	Female	329	65.9
Grade	Freshmen	227	45.5
	Sophomores	134	26.9
	Juniors	122	24.4
	Seniors	16	3.2
Daily duration	1-3h	140	28.1
	3-6h	234	46.9
	More than6h	125	25.1

### Measurement instruments

The measurement instruments consisted of two parts. The first part collected participants’ demographic information, while the second part gathered self-reported data on each construct. Established and validated scales were adopted to assess these constructs, with appropriate modifications to fit the specific research context and objectives. This approach ensured that the measures accurately reflected participants’ conditions within the study setting.

In addition to basic demographic information, the measurement instruments included six key constructs: SMU, SN, ESE, EA, PBC, and EI. Compared with the seven-point Likert scale, the five-point Likert scale offers notable advantages in improving reliability and validity [77], reducing bias, and enhancing the sensitivity of statistical analyses [78]. Accordingly, all constructs in this study were measured using a five-point Likert scale, ranging from (1) “strongly disagree” to (5) “strongly agree.”

The SMU scale was adapted from Zhang, Abbas [79]. It was designed to measure the extent to which culinary arts students use social media in restaurant entrepreneurship. The scale consists of five items (e.g., “Social media provides me with opportunities to learn about restaurant entrepreneurship knowledge”). The Cronbach’s alpha coefficient for this scale was 0.869, indicating good reliability. Notably, the items emphasize learning-oriented and entrepreneurship-related uses of social media, rather than general recreational engagement, ensuring consistency between the conceptual definition and the operationalization of SMU in this study.

The SN scale was adapted from Nguyen and Nguyen [23]. It was employed to assess the social pressure or support perceived by culinary arts students from family, friends, and peers when making decisions about restaurant entrepreneurship. The scale comprises three items (e.g., “If I choose to pursue restaurant entrepreneurship, my family will support me”). The Cronbach’s alpha was 0.861, indicating good reliability.

The PBC scale was adapted from Tchokoté, Bawack [80]. It was used to measure the extent to which culinary arts students perceive themselves as capable of managing and controlling the process of restaurant entrepreneurship. The scale consists of six items (e.g., “It would be easy for me to start and run a restaurant business”). The Cronbach’s alpha was 0.938, indicating excellent reliability.

The ESE scale was adapted from Duong [81] and was employed to measure the level of self-efficacy perceived by culinary arts students in restaurant entrepreneurship. The scale comprises five items (e.g., “I have strong creativity and innovative ability”). The Cronbach’s alpha was 0.903, indicating high reliability.

The EI scale was adapted from Duong [81] and was designed to measure the extent of culinary arts students’ willingness to engage in entrepreneurial activities in the restaurant sector. The scale consists of six items (e.g., “I am willing to do whatever it takes to become a restaurant entrepreneur”). The Cronbach’s alpha was 0.906, indicating high reliability.

The EA scale was adapted from Tchokoté, Bawack [80] and was used to assess culinary arts students’ attitudes toward entrepreneurship in the restaurant sector. The scale comprises five items (e.g., “For me, the benefits of becoming a restaurant entrepreneur outweigh the drawbacks”). The Cronbach’s alpha was 0.867, indicating good reliability.

### Data analysis

This study employed a two-stage analytical approach to test the hypotheses and construct the predictive model. First, Partial Least Squares–Structural Equation Modeling was used to identify the linear relationships between exogenous and endogenous constructs. Partial Least Squares-Structural Equation Modeling offers substantial advantages in small sample sizes, non-normal data distributions, exploratory research, and complex models [76]. With a sample size 499 and a model comprising six constructs and 30 items, this study qualifies as an exploratory analysis of a complex model [82]. Therefore, Partial Least Squares-Structural Equation Modeling was appropriate for the data analysis. However, structural equation modeling has inherent limitations in capturing nonlinear and non-compensatory relationships, critical for understanding social media’s influence on culinary arts students’ EI.

To address these limitations, the second stage of this study incorporated ANN. ANN enable non-compensatory models to capture linear and nonlinear relationships, enhancing predictive accuracy [83]. Moreover, prior studies have highlighted the robustness of ANN in handling complex data patterns and prediction tasks [84]. By integrating SEM with ANN, this study increased the depth and precision of the data analysis, allowing for a more comprehensive understanding of how social media influences the EI of culinary arts students and the relative importance of the contributing factors.

### Ethics statement

This study was reviewed and approved by the Human Research Ethics Committee of Sichuan Tourism University (Approval No. SCTU-2025-01-0001). The study complies with the National Statement on Ethical Conduct in Human Research (2007). Written informed consent was obtained from all participants prior to their involvement in the research.

## Results

The researchers employed different statistical methods to develop and validate the study’s findings. Hair [85] distinguishes between two generations of statistical approaches. First-generation techniques, such as factor and regression analysis, dominated early research and were widely applied. Since the 1990s, more advanced multivariate techniques, such as SEM, have emerged as the dominant methods of second-generation statistics [86]. SEM is generally classified into two types: covariance-based SEM and variance-based SEM. Given the complexity of the present model—with six constructs, 30 items, and eight relationships—Partial Least Squares-Structural Equation Modeling was deemed appropriate for analysis [85]. Accordingly, Smart Partial Least Squares 4.0 was used to assess this study’s measurement and structural models.

### Measurement model

The evaluation of the measurement model followed established guidelines.

Step 1: Indicator reliability. The outer loadings of all indicators should be ≥ 0.70 [85].

Step 2: Internal consistency reliability. Two tests were used: Cronbach’s alpha (α) and composite reliability (CR). For both measures, the threshold value should be ≥ 0.70 [87].

Step 3: Validity.

(1) *Convergent validity*: The Average Variance Extracted (AVE) should be ≥ 0.50 [88].
(2) *Discriminant validity*: Two methods were applied to assess discriminant validity:

The Fornell–Larcker criterion [88].

The Heterotrait–Monotrait (HTMT) ratio of correlations [89].

First, following Hair [85] recommendations, the outer loadings of the items should exceed 0.70. In this study, all 30 indicators had outer loadings greater than 0.70.

Second, internal consistency reliability and convergent validity were assessed using Cronbach’s alpha, CR, and AVE. As shown in Table 2, all values satisfied the minimum criteria for indicator reliability and internal consistency reliability. Moreover, the AVE for all constructs was ≥ 0.50, confirming adequate convergent validity.

**Table 2:** Reliability and average variance extracted.

Constructs	Items	Outer loadings	α	CR	AVE
EA	EA1	0.848	0.867	0.876	0.656
	EA2	0.873			
	EA3	0.791			
	EA4	0.841			
	EA5	0.701			
EI	EI1	0.775	0.906	0.910	0.683
	EI2	0.872			
	EI3	0.814			
	EI4	0.893			
	EI5	0.829			
	EI6	0.767			
ESE	ESE1	0.801	0.903	0.905	0.721
	ESE2	0.848			
	ESE3	0.862			
	ESE4	0.881			
	ESE5	0.850			
PBC	PBC1	0.804	0.938	0.939	0.764
	PBC2	0.903			
	PBC3	0.915			
	PBC4	0.900			
	PBC5	0.893			
	PBC6	0.824			
SMU	SMU1	0.719	0.869	0.881	0.659
	SMU2	0.850			
	SMU3	0.858			
	SMU4	0.864			
	SMU5	0.799			
SN	SN1	0.875	0.861	0.865	0.783
	SN2	0.890			
	SN3	0.888			

Third, Table 3 presents the correlation matrix used to assess discriminant validity based on the Fornell–Larcker criterion. According to Hair [76], the square root of the AVE for each construct should be greater than its highest correlation with any other construct in the model. The results clearly met this standard, confirming adequate discriminant validity.

**Table 3:** Discriminant validity (Fornell-Larcker criteria).

Constructs	EA	EI	ESE	PBC	SMU	SN
EA	**0.810**					
EI	0.781	**0.826**				
ESE	0.591	0.626	**0.849**			
PBC	0.558	0.625	0.667	**0.874**		
SMU	0.351	0.302	0.344	0.303	**0.812**	
SN	0.453	0.426	0.406	0.372	0.587	**0.885**

Note: The bold values on the diagonal represent the square root of the Average Variance Extracted for each construct.

The HTMT, proposed by Henseler, Ringle [89], is a criterion for assessing discriminant validity. The HTMT is defined as the ratio of the average heterotrait–heteromethod correlations to the average monotrait–heteromethod correlations. Heterotrait–heteromethod correlations refer to correlations between indicators across different constructs, whereas monotrait–heteromethod correlations refer to correlations between indicators within the same construct. This study’s HTMT values were calculated using Smart Partial Least Squares (see Table 4). All the HTMT values fell within the acceptable threshold of ≤ 0.85 [89], confirming discriminant validity.

**Table 4:** Discriminant validity (The Heterotrait–Monotrait criteria).

Constructs	EA	EI	ESE	PBC	SMU	SN
EA						
EI	0.811					
ESE	0.662	0.692				
PBC	0.618	0.677	0.723			
SMU	0.406	0.339	0.387	0.332		
SN	0.521	0.479	0.458	0.411	0.674	

### Structural model

The structural model was assessed following the recommendations of Hair [76] using the following steps:

(1) Collinearity assessment: Variance Inflation Factor (VIF) values should be less than 3.3 to indicate the absence of multicollinearity issues.
(2) Significance and relevance of structural relationships: Path coefficients were evaluated based on their significance levels, with *p* < 0.05 considered statistically significant.
(3) Coefficient of determination (R²): The model’s explanatory power was assessed using R² values, with threshold levels of 0.190 (weak), 0.333 (moderate), and 0.670 (substantial).

First, collinearity was assessed using the VIF. A VIF value ≥ 3.3 indicates potential collinearity issues. As shown in Table 5, all VIF values were below the acceptable threshold of 3.3 [76]. Therefore, no collinearity problems were detected.

**Table 5:** VIF.

Constructs	EA	EI	ESE	PBC	SMU	SN
EA		1.625				
EI						
ESE	1					
PBC		1.497				
SMU		1.555	1	1		1
SN		1.728				

Second, Table 6 presents the path coefficients (β values) for the relationships among the constructs in the model. The significance of the path coefficients was assessed using the bootstrapping procedure in Partial Least Squares. The *t*-values and *p*-values were used to test whether the path coefficients (β) were statistically significant at the 5% level. A 5% significance level requires *p* < 0.05 and *t* > 1.96. Among *t*he direct predictors of EI, EA exerted the strongest effect (β = 0.667, *t* = 20.964, *p* = 0.000), followed by PBC (β = 0.244, *t* = 7.412, *p* = 0.000). In addi*t*ion, SMU significan*t*ly predicted ESE (β = 0.344, *t* = 7.458, *p* = 0.000), PBC (β = 0.303, *t* = 7.364, *p* = 0.000), and SN (β = 0.587, *t* = 14.641, *p* = 0.000). However, SMU (β = –0.038, *t* = 0.847, *p* = 0.397) and SN (β = 0.055, *t* = 1.419, *p* = 0.156) did no*t* significan*t*ly predict EI.

**Table 6:** Hypothesis testing.

Hypotheses	β	T statistics	P values	Results
EA → EI	0.667	20.964	0.000	Supported
ESE → EA	0.591	14.454	0.000	Supported
PBC → EI	0.244	7.412	0.000	Supported
SMU → EI	−0.038	0.847	0.397	Not Supported
SMU → ESE	0.344	7.458	0.000	Supported
SMU → PBC	0.303	7.364	0.000	Supported
SMU → SN	0.587	14.641	0.000	Supported
SN → EI	0.055	1.419	0.156	Not Supported

Third, the coefficient of determination (R²) represents the proportion of variance in an endogenous construct explained by its associated exogenous constructs [76]. Values of approximately 0.67 are considered substantial, around 0.33 moderate, and around 0.19 weak. As shown in Table 7, SMU, SN, PBC, EA, and ESE explained 70.7% of the variance in EI, indicating substantial explanatory power.

**Table 7:** R^2^.

Constructs	R^2^
EA	0.349
EI	0.707
ESE	0.118
PBC	0.092
SN	0.345

### Mediation analysis

To assess the mediating roles of SN, PBC, ESE, and EA in the relationship between SMU and EI among culinary arts students, this study employed the bootstrapping procedure in Partial Least Squares-Structural Equation Modeling [90]. Both direct and indirect effects were examined to determine the type and magnitude of mediation. As shown in Table 8, PBC (β = 0.074, *t* = 5.100, *p* = 0.000) and the combined mediation of ESE and EA (β = 0.135, *t* = 5.542, *p* = 0.000) exerted significant indirect effects in the relationship between SMU and EI. However, the direct effect of SMU on EI (β = –0.038, *t* = 0.847, *p* = 0.397) was insignificant. These findings indicate that PBC, ESE, and EA fully mediate the relationship between SMU and EI among culinary arts students. Finally, SN (β = 0.032, *t* = 1.385, *p* = 0.166) showed no significant direct or indirect effects, suggesting that SN do not mediate this relationship.

**Table 8:** Mediation analysis.

Relation	Indirect effect	T	P	Direct Effect	T	P	Type
Mediation effect of PBC							
SMU → PBC → EI	0.074	5.100	0.000	−0.038	0.847	0.397	FM
Mediation effect of ESE and EA							
SMU → ESE → EA → EI	0.135	5.542	0.000	−0.038	0.847	0.397	FM
Mediation effect of SN							
SMU → SN → EI	0.032	1.385	0.166	−0.038	0.847	0.397	NM

Note: FM – Full Mediation; NM- Non-Mediation

### Artificial neural network analysis

Given the potential nonlinear relationships between exogenous and endogenous variables, this study used the significant factors from the SEM path analysis as input neurons in the ANN model. Specifically, a feed-forward multilayer perceptron (MLP) architecture with one hidden layer was adopted, where SEM predictors served as input neurons and EI was specified as the output neuron. The use of SEM predictors as ANN inputs was intended to ensure theoretical consistency while allowing the ANN to explore potential nonlinear and non-compensatory relationships beyond linear path estimation. ANN are robust to noise, outliers, and relatively small sample sizes, and they can be applied to non-compensatory models in which a decrease in one factor does not need to be offset by an increase in another. The ANN analysis was implemented using IBM SPSS Neural Network Module. ANN algorithms can capture linear and nonlinear relationships and do not require data to follow a normal distribution [91]. Through iterative training, the model learns using the feed-forward back-propagation algorithm to predict outcomes [92]. Multilayer perceptrons with sigmoid activation functions were employed in both the input and hidden layers [93]. Multiple learning iterations were conducted to minimize errors and improve predictive accuracy [94]. This study used 70% of the sample for training, while the remaining data were reserved for testing. A ten-fold cross-validation procedure was performed to avoid potential overfitting, and the root mean square error was obtained [95]. As shown in Table 9, the average root mean square error values for the training and testing processes were 0.1357 and 0.1351, respectively, indicating stable and comparable predictive performance across training and testing samples.

**Table 9:** Root mean square of error values.

Training			Testing			Total samples
N	SSE	RMSE	N	SSE	RMSE	
336	6.1723	0.1355	163	2.9341	0.1342	499
354	6.6038	0.1366	145	2.2223	0.1238	499
229	5.5375	0.1555	160	2.8686	0.1339	389
337	5.6868	0.1299	162	2.3674	0.1209	499
346	6.2758	0.1347	153	2.4854	0.1275	499
361	6.5236	0.1344	138	2.9111	0.1452	499
342	6.0410	0.1329	157	3.3094	0.1452	499
349	6.1328	0.1326	150	2.5984	0.1316	499
353	5.8566	0.1288	146	3.1219	0.1462	499
351	6.5326	0.1364	148	2.9999	0.1424	499
Mean	6.1363	0.1357	Mean	2.7818	0.1351	
Sd		0.0074	Sd		0.0093	

Note: N: number of samples; SSE: sum of squares of error; RMSE: root mean square of error.

A sensitivity analysis was conducted to evaluate the predictive power of each input neuron (Table 10). The normalized importance of each neuron was obtained by dividing its relative importance by the highest observed importance and expressing the result as a percentage [96]. The results revealed that EA was the most important predictor, with a normalized importance of 100%. This was followed by PBC (25.4%), ESE (20.7%), SMU (17.2%), and SN (13.1%).

**Table 10:** Sensitivity analysis.

ANN	EA	ESE	PBC	SMU	SN
ANN 1	1.000	0.237	0.284	0.162	0.138
ANN2	1.000	0.267	0.262	0.175	0.165
ANN3	1.000	0.190	0.265	0.154	0.137
ANN4	1.000	0.253	0.177	0.183	0.160
ANN5	1.000	0.167	0.318	0.113	0.095
ANN6	1.000	0.164	0.263	0.160	0.109
ANN7	1.000	0.221	0.209	0.146	0.142
ANN8	1.000	0.164	0.315	0.257	0.153
ANN9	1.000	0.235	0.196	0.158	0.086
ANN10	1.000	0.173	0.251	0.208	0.124
Mean importance	1.000	0.207	0.254	0.172	0.131
Normalised importance (%)	100.0%	20.7%	25.4%	17.2%	13.1%

## Discussion

Guided by three core research questions, this study systematically examines the mechanisms and predictive efficacy through which SMU shapes the EI of university students majoring in culinary arts. Building on the principal findings and drawing on SCT and the TPB, the discussion develops an in-depth analysis along four dimensions: theoretical mechanisms, inter-variable relationships, predictor rankings, and theoretical contributions.

The findings reveal that SMU does not significantly affect the EI of culinary arts students (*p* = 0.397). This result contrasts with some prior studies [56], yet individual cognitive processes and the distinctive characteristics of the culinary profession can explain it. According to SCT, external sources of information do not automatically translate into behavioral intentions; instead, they must be processed through mediating mechanisms such as self-efficacy, outcome expectations, and PBC [16]. For culinary students, EI is more strongly rooted in their perceptions of skill maturity, hands-on experience, and mastery of operational processes, rather than solely in entrepreneurial information provided through social media. Thus, even though social media may offer examples of restaurant entrepreneurship or industry-related knowledge, the lack of immediate feedback and offline practical support means that such informational stimuli are insufficient to drive the formation of EI directly.

The results demonstrate that EA and PBC positively affect EI, consistent with prior research [68,73], emphasizing that these factors significantly strengthen EI by enhancing students’ confidence. This finding is highly aligned with the core mechanisms of the TPB, which identifies EA and PBC as key predictors of behavioral intention [59]. However, our results differ from a more petite body of studies that found the influence of EA or PBC on EI weak or insignificant [97,98]. Such discrepancies may stem from differences in research populations. For example, Batz Liñeiro, Romero Ochoa [98] examined actual entrepreneurs, many of whom were necessity-driven entrepreneurs, such as migrants or individuals from disadvantaged groups, for whom entrepreneurship was often the only option due to unemployment, discrimination, or economic crises. In contrast, culinary arts students represent a group that proactively chooses their academic and career path based on personal interest and professional skills. Their EI are shaped by a sense of professional identity and career vision, whereby positive attitudes toward restaurant entrepreneurship directly enhance their intentions by highlighting the attractiveness and value of entrepreneurial behavior. At the same time, their PBC—strengthened through specialized training—reduces anticipated entrepreneurial barriers, boosts confidence in success, and consequently increases EI.

The results indicate that SN were insignificant as a mediator between SMU and EI or as a direct predictor of EI (*p* > 0.1). This finding deviates from the expectations of the TPB [59], yet it can be reasonably explained within the specific learning and decision-making context of culinary arts students.. Among members of Generation Z, particularly culinary arts students, career cognition increasingly emphasizes perceived personal competence and task feasibility. Rather than focusing on “what others think,” these students emphasize “whether I can perform well.” From a broader perspective, social expectations may influence entrepreneurial intention indirectly by being internalized into individuals’ confidence and perceived feasibility, rather than acting as a direct motivational force. In the Chinese educational and cultural context, social expectations are often conveyed implicitly rather than through explicit normative pressure. In this context, social expectations from family members, peers, or instructors may not function as a direct normative pressure. Instead, such social influences are more likely to be internalized into students’ confidence and perceived feasibility through learning and feedback processes, thereby indirectly shaping entrepreneurial intention and weakening the direct effect of subjective norms. In this study, these findings point to specific boundary conditions under which the effects of subjective norms on entrepreneurial intention are attenuated, namely the practice-oriented nature of culinary arts education, the emphasis on skill mastery and perceived task feasibility, and the implicit transmission of social expectations in the Chinese educational context.

Although SMU did not have a direct effect on EI, it exerted an indirect influence through two mediating pathways: PBC (β = 0.074, *p* < 0.001) and the combined mechanism of ESE and EA (β = 0.135, *p* < 0.001). This finding is consistent with SCT’s three-stage model of “environment–cognition–behavior,” suggesting that social media, as a source of information and social input, can empower individuals’ internal cognition and stimulate entrepreneurial motivation [99]. The dual-path mechanism of ESE and PBC is particularly noteworthy: on the one hand, social media enhances students’ sense of control by disseminating entrepreneurial knowledge and success stories; on the other hand, it strengthens their confidence in accomplishing entrepreneurial tasks, which in turn fosters more positive EA and ultimately drives the formation of EI.

The ANN analysis revealed that EA had the highest predictive importance among all variables (normalized importance = 100%), far exceeding PBC (25.4%), ESE (20.7%), SMU (17.2%), and SN (13.1%). This ranking reinforces the “attitude-dominant” structural logic of the TPB [33]. From a theoretical perspective, TPB posits that attitude represents individuals’ overall evaluative orientation toward a behavior and often serves as the most proximal psychological driver of intention when individuals perceive the behavior as personally meaningful and volitional. For culinary arts students, restaurant entrepreneurship is closely tied to personal interest, professional identity, and value realization, making evaluative judgments about desirability and attractiveness more influential than external constraints or social pressure. Accordingly, entrepreneurial attitude functions as a central cognitive integrator that synthesizes information from perceived feasibility and self-efficacy into a general willingness to engage in entrepreneurial action, which explains its dominant predictive role in the ANN results. Although social media did not exhibit a direct effect, its moderate predictive power in the ANN ranking indicates its important role as a “cognitive activation source.” While social media alone may not directly drive intention, it indirectly enhances the overall momentum of intention formation by strengthening key psychological variables.

### Theoretical implications

First, this study is the first to examine the EI of culinary arts students from the perspective of SMU. Previous research on EI among university students has primarily focused on factors such as individual traits, educational and economic environments, and sociocultural influences. In contrast, SMU—as a behavioral variable that reflects patterns of information acquisition and social interaction—has received limited attention in this domain. By systematically analyzing its impact on the EI of culinary arts students, this study fills a gap in the literature on specialized disciplines. It provides a novel theoretical lens for understanding the motivational drivers of entrepreneurship.

Second, this study employed a hybrid SEM–ANN approach to capture the linear and nonlinear relationships and the non-compensatory effects between SMU and EI. This methodological integration provides a novel perspective for research on the EI of culinary arts students. Beyond enhancing the accuracy of predictions, the combined method offers precise empirical evidence to inform entrepreneurship education in universities and policymaking by government institutions. Moreover, it serves as a methodological reference for future studies examining SMU’s psychological and behavioral impacts, offering broad academic value.

Third, this study integrates SCT and the TPB to explore the influence of SMU on students’ EI. Specifically, by combining the core constructs of the two theories—SN, PBC, ESE, and EA—this study developed a comprehensive model that examined the direct effects of SMU and revealed its indirect influence through multiple mediating pathways. This theoretical integration uncovers the psychological and behavioral drivers of EI among culinary arts students. It provides a new perspective for understanding the relationship between SMU and EI.

Fourth, the study’s inconsistent findings—such as the non-significant direct effects of SMU and SN on EI—open new avenues for future research. These divergences enrich the theoretical understanding of the relationship between SMU and the EI of culinary arts students, highlighting boundary conditions and contextual factors that may shape entrepreneurial cognition. In this study, boundary conditions refer to the contextual and disciplinary circumstances under which established theoretical relationships may weaken or operate through indirect rather than direct pathways. Specifically, the skill-intensive and practice-oriented nature of culinary arts education, together with culturally implicit forms of social influence, shapes how entrepreneurial intention is formed and limits the direct applicability of normative pressures. The study identifies these nuances and provides important academic insights and practical directions for subsequent research.

### Practical implications

This study investigated the impact of SMU on the EI of culinary arts students and found that, although SMU did not directly influence EI, it indirectly affected intention through mediating variables such as PBC, ESE, and EA. These findings not only enrich the application of social media research in student entrepreneurship but also carry important practical value for policymakers, educational institutions, educators, and culinary arts students with entrepreneurial aspirations.

Given that SN do not directly influence the EI of culinary arts students, while PBC, ESE, and EA play decisive roles, policymakers should focus on empowering individuals rather than merely fostering a general entrepreneurial climate. Specifically, policies should strengthen students’ practical abilities and entrepreneurial confidence. For example, a dedicated “Culinary Entrepreneurship Training Subsidy” program could provide financial support for students who participate in restaurant kitchen rotations or complete standardized dish development projects, enabling them to gain hands-on experience and enhance their control over essential entrepreneurial skills. In addition, initiatives such as a “Youth Culinary Entrepreneurship Competition” could be organized, offering media exposure and access to entrepreneurial resources for outstanding participants, thereby stimulating students’ ESE and motivation.

Given that PBC was found to significantly influence the EI of culinary arts students, educational institutions and educators should prioritize strengthening students’ PBC. Specifically, they should focus on cultivating students’ entrepreneurial competencies and enhancing their ability to manage entrepreneurial processes. For instance, institutions could introduce a “Culinary Entrepreneurship Skills Development” course, requiring students to acquire restaurant entrepreneurship skills and industry insights through case studies and hands-on practice. Moreover, institutions could establish an “Entrepreneurial Competency Growth Portfolio,” which tracks students’ progress using both online engagement data from social media (e.g., likes and comments on culinary creations) and offline performance assessments (e.g., time-limited menu design and cost estimation tasks). This would reinforce students’ confidence in their entrepreneurial capabilities. Additionally, educators could provide personalized mentoring tailored to students’ specific weaknesses. For example, students lacking knowledge of supply chain management could be guided with supplier-matching case studies and tutorials on inventory optimization.

As an EA was identified as the strongest predictor of EI, culinary arts students should maintain optimism to strengthen their intentions and increase the likelihood of entrepreneurial success. Specifically, students can reinforce their EA through coursework and participation in restaurant entrepreneurship projects. For example, they may proactively enroll in restaurant entrepreneurship management courses or attend industry expert seminars to foster cognitions such as “entrepreneurship brings more benefits than drawbacks” and “restaurant entrepreneurship is highly attractive.” In addition, culinary arts students can engage in campus-based restaurant entrepreneurship initiatives or culinary competitions, using these practical experiences to strengthen their belief that “entrepreneurship will lead to success” and to cultivate an entrepreneurial spirit characterized by optimism and perseverance.

### Limitations and future research

While this study provides valuable insights into the impact of SMU on the EI of culinary arts students, several limitations should be acknowledged, offering opportunities for future research. First, the data were collected using a cross-sectional design, which does not capture how the influence of social media on EI may evolve. Future studies could adopt longitudinal designs to examine SMU’s temporal dynamics and long-term effects on EI. Second, this study primarily employed a quantitative research design. Although quantitative methods allow for hypothesis testing and generalization, they may not fully capture the nuanced mechanisms through which different types of SMU affect EI. Future research could adopt mixed-method approaches, incorporating qualitative techniques such as interviews or focus groups, to provide richer contextual insights and explanatory depth. Third, the sample in this study was gender-imbalanced and drawn exclusively from Sichuan Province. In addition, the use of a snowball sampling approach may further constrain the representativeness of the sample. Given that gender and regional differences may influence students’ psychological, physiological, and social behaviors, the findings should be interpreted as context-dependent and may not fully represent the broader population of culinary arts students. Replicating this study with more diverse, gender-balanced, and geographically representative samples would help examine the robustness and boundary conditions of the proposed relationships and strengthen the validity and generalizability of the results.

## Conclusion

This study aimed to investigate the mechanisms through which SMU influences the EI of culinary arts students. Drawing on SCT and the TPB, the study clarified how SMU affects EI through SN, PBC, ESE, and EA. Data were collected from 499 culinary arts students in Sichuan Province, China. Using a SEM–ANN hybrid approach, the findings revealed that social media use did not directly influence EI; instead, it had positive indirect effects through mediators such as PBC, ESE, and EA. The ANN analysis further uncovered nonlinear dynamics among the variables, showing that EA was the strongest direct predictor of EI among culinary arts students. By adopting a behavioral perspective on SMU, this study makes a novel contribution to the literature on entrepreneurship in non-traditional disciplines. It addresses a theoretical gap in understanding the EI of culinary arts students. Methodologically, integrating SEM and ANN enhanced prediction accuracy and provided a new methodological reference for entrepreneurship research. Theoretically, this study combined the TPB and SCT to develop a more systematic cognitive model of EI, extending the theoretical boundaries of entrepreneurship research. The inconsistent findings—such as the non-significant role of SN and the absence of direct effects of SMU—provide valuable future research directions. These findings have practical relevance for addressing students’ entrepreneurial needs and offer theoretical support for designing more targeted and effective entrepreneurship education interventions. Nonetheless, limitations such as gender imbalance, single regional focus, and cross-sectional design may restrict the generalizability and depth of the results. Future research should address these limitations by employing longitudinal designs, broader and more diverse samples, and mixed-method approaches to enhance the comprehensiveness and practical utility of the findings.

## Supporting information

S1 Data Raw survey data. This file contains the de-identified raw data collected for this study and constitutes the minimal dataset required to replicate all analyses reported in the manuscript.
(XLSX)

## References

[1] Boucif SA. Extending the theory of planned behavior in predicting entrepreneurial intention among university students: The role of perceived relational support. Inter J Manag Edu. 2025;23(2):101168.

[2] Tekic A, Tsyrenova E. Drivers and constraints of students’ entrepreneurial intentions across cultural contexts: a neo-configurational perspective. Intern J Manag Edu. 2024;22(3):100996. doi: 10.1016/j.ijme.2024.100996

[3] Wu X, Tian Y. Predictors of entrepreneurship intention among students in vocational colleges: a structural equation modeling approach. Front Psychol. 2022;12:797790. doi: 10.3389/fpsyg.2021.797790 35095683 PMC8790017

[4] Guo H. Research on the “2 + 1” talent training model in higher vocational railway colleges under the “Four Platforms and One Meeting” linkage mechanism. J Modern Educ Theor Pract. 2024;1(1). doi: 10.70767/jmetp.v1i1.147

[5] Liu Z, Zhang M, Guo Y, Mao T, Deng S, Li Y. Entrepreneurship education stimulates entrepreneurial intention of college students in China: A dual-pathway model. The International Journal of Management Education. 2025;23(2):101107. doi: 10.1016/j.ijme.2024.101107

[6] Olanrewaju A-ST, Hossain MA, Whiteside N, Mercieca P. Social media and entrepreneurship research: a literature review. Inter J Inform Manag. 2020;50:90–110. doi: 10.1016/j.ijinfomgt.2019.05.011

[7] Purnama Y, Asdlori A. The role of social media in students’ social perception and interaction: implications for learning and education. Technol Soc Persp. 2023;1(2):45–55. doi: 10.61100/tacit.v1i2.50

[8] Jian L, Peng C, Linyan M. Understanding the influence of social media on university students’ communication skills in digital information environment. Profesional de la inform. 2025;33(6).

[9] Kolhar M, Kazi RNA, Alameen A. Effect of social media use on learning, social interactions, and sleep duration among university students. Saudi J Biol Sci. 2021;28(4):2216–22. 33911938 10.1016/j.sjbs.2021.01.010 PMC8071811

[10] Asghar A, Haneef M, Dhillon NR, Jan R. The influence of social media use on academic performance: exploring the role of cognitive load, self-regulation, and motivation among student. Rev Appl Manag Soc Sci. 2024;7(4):725–40. doi: 10.47067/ramss.v7i4.409

[11] Ghouse SM, Barber III D, Alipour K. Shaping the future entrepreneurs: influence of human capital and self-efficacy on entrepreneurial intentions of rural students. Inter J Manag Educ. 2024;22(3):101035. doi: 10.1016/j.ijme.2024.101035

[12] Fu X, Yan T, Tian Y, Niu X, Xu X, Wei Y, et al. Exploring factors influencing students’ entrepreneurial intention in vocational colleges based on structural equation modeling: evidence from China. Front Psychol. 2022;13:898319. doi: 10.3389/fpsyg.2022.898319 35747685 PMC9211024

[13] Chadha P, Upadhaya G, Devi N. Exploring the nexus of personality traits, self-efficacy, and entrepreneurial intention: a study of university students. Inter J Manag Edu. 2025;23(2):101136. doi: 10.1016/j.ijme.2025.101136

[14] Niu X, Niu Z, Wang M, Wu X. What are the key drivers to promote entrepreneurial intention of vocational college students? An empirical study based on structural equation modeling. Front Psychol. 2022;13:1021969. doi: 10.3389/fpsyg.2022.1021969 36389516 PMC9650398

[15] Christensen BT, Arendt KM, McElheron P, Ball LJ. The design entrepreneur: How adaptive cognition and formal design training create entrepreneurial self-efficacy and entrepreneurial intention. Design Stud. 2023;86:101181. doi: 10.1016/j.destud.2023.101181

[16] Bandura A. Social foundations of thought and action. Englewood Cliffs, NJ. 1986.

[17] Chen M, Cao Y, Liang Y. Determinants of open government data usage: integrating trust theory and social cognitive theory. Govern Inform Quart. 2023;40(4):101857. doi: 10.1016/j.giq.2023.101857

[18] Schunk DH, DiBenedetto MK. Motivation and social cognitive theory. Contemp Edu Psychol. 2020;60:101832.

[19] Bandura A. Social cognitive theory: an agentic perspective. Annu Rev Psychol. 2001;52:1–26. doi: 10.1146/annurev.psych.52.1.1 11148297

[20] Liao YK, Nguyen VHA, Caputo A. Unveiling the role of entrepreneurial knowledge and cognition as antecedents of entrepreneurial intention: a meta-analytic study. Inter Entrepren Manag J. 2022;18(4):1623–52.

[21] Sutrisno S, Siminto S, Syamsuri S, Junianto P, Pramono SA. The influence of ChatGPT usage and entrepreneurship education on students’ entrepreneurial intentions with innovative behaviour as a mediating variable : the perspective of social cognitive and experiential learning theory. J Kependidikan Penelit Kaji Kepustakaan Bid Pendidik Pengajaran. 2024;10(3):906. doi: 10.33394/jk.v10i3.12375

[22] Liu M. Learning goal orientation, social capital, and entrepreneurial intentions: a multigroup analysis within entrepreneurship programs. Inter J Manag Edu. 2024;22(3):101043. doi: 10.1016/j.ijme.2024.101043

[23] Nguyen GN, Nguyen TK. Entrepreneurial passion of business and technical students: the roles of subjective norms, entrepreneurial education, entrepreneurial self-efficacy, and risk propensity. Inter J Manag Edu. 2024;22(3):101012. doi: 10.1016/j.ijme.2024.101012

[24] Valdez-Juárez LE, García Pérez-de-Lema D. Creativity and the family environment, facilitators of self-efficacy for entrepreneurial intentions in university students: case ITSON Mexico. Inter J Manag Edu. 2023;21(1):100764. doi: 10.1016/j.ijme.2023.100764

[25] Ajzen I. The theory of planned behavior. Organiz Behav Human Decision Process. 1991;50(2):179–211. doi: 10.1016/0749-5978(91)90020-t

[26] Boubker O. Does religion raise entrepreneurial intention and behavior of Muslim university students? An extension of Ajzen’s theory of planned behavior (TPB). Inter J Manag Edu. 2024;22(3):101030. doi: 10.1016/j.ijme.2024.101030

[27] Relente ARR, Capistrano EPS. Innovation self-efficacy, theory of planned behavior, and entrepreneurial intentions: the perspective of young Filipinos. Asia Pacific Manag Rev. 2025;30(3):100350. doi: 10.1016/j.apmrv.2024.100350

[28] Yasir N, Babar M, Mehmood HS, Xie R, Guo G. The environmental values play a role in the development of green entrepreneurship to achieve sustainable entrepreneurial intention. Sustainability. 2023;15(8):6451. doi: 10.3390/su15086451

[29] Slomski VG, Tavares de Souza Junior AV, Lavarda CEF, Simão Kaveski ID, Slomski V, Frois de Carvalho R, et al. Environmental factors, personal factors, and the entrepreneurial intentions of university students from the perspective of the theory of planned behavior: contributions to a sustainable vision of entrepreneurship in the business area. Sustainability. 2024;16(13):5304. doi: 10.3390/su16135304

[30] Garcez A, Franco M, Silva R. The influence of the pillars of digital academic entrepreneurship on university students’ entrepreneurial intention. EJIM. 2023;28(2):210–34. doi: 10.1108/ejim-01-2023-0051

[31] Ferri L, Spanò R, Theodosopoulos G, Tsitsianis N. University education and entrepreneurial intentions of European students: insights into the theory of planned behaviour complemented by skills. Stud Higher Edu. 2023;49(9):1625–39. doi: 10.1080/03075079.2023.2272161

[32] Maheshwari G, Kha KL. Investigating the relationship between educational support and entrepreneurial intention in Vietnam: The mediating role of entrepreneurial self-efficacy in the theory of planned behavior. Inter J Manag Edu. 2022;20(2):100553. doi: 10.1016/j.ijme.2021.100553

[33] Tchokoté ID, Bawack R, Nana A. Attitude over norms: reevaluating the dominance of attitude in shaping entrepreneurial intentions among higher education students in Global South countries. Inter J Manag Edu. 2025;23(2):101129.

[34] Nwosu HE, Obidike PC, Ugwu JN, Udeze CC, Okolie UC. Applying social cognitive theory to placement learning in business firms and students’ entrepreneurial intentions. Inter J Manag Edu. 2022;20(1):100602. doi: 10.1016/j.ijme.2022.100602

[35] Bell R. Predicting entrepreneurial intention across the university. Educ Train. 2019;61(7/8):815–31. doi: 10.1108/et-05-2018-0117

[36] Çelik AK, Yıldız T, Aykanat Z, Kazemzadeh S. The impact of narrow personality traits on entrepreneurial intention in developing countries: a comparison of Turkish and Iranian undergraduate students using ordered discrete choice models. Euro Res Manag Business Econ. 2021;27(1):100138. doi: 10.1016/j.iedeen.2020.100138

[37] Villanueva-Flores M, Hernández-Roque D, Díaz-Fernández M, Bornay-Barrachina M. Exploring the mediation role of perceived behavioural control and subjective norms in the relationship between psychological capital and entrepreneurial intention of university students. Inter J Manag Educ. 2023;21(3):100865. doi: 10.1016/j.ijme.2023.100865

[38] Pelegrini GC, Moraes GHSM d HSM d. Does gender matter? A university ecosystem, self-efficacy and entrepreneurial intention analysis in Brazilian universities. Gender Manag. 2021;37(2):271–86.

[39] António Porfírio J, Augusto Felício J, Carrilho T, Jardim J. Promoting entrepreneurial intentions from adolescence: the influence of entrepreneurial culture and education. J Business Res. 2023;156:113521. doi: 10.1016/j.jbusres.2022.113521

[40] Schlaegel C, Engle RL, Richter NF, Taureck PC. Personal factors, entrepreneurial intention, and entrepreneurial status: a multinational study in three institutional environments. J Int Entrep. 2021;19(3):357–98. doi: 10.1007/s10843-021-00287-7

[41] Shahzad MF, Khan KI, Saleem S, Rashid T. What factors affect the entrepreneurial intention to start-ups? The role of entrepreneurial skills, propensity to take risks, and innovativeness in open business models. J Open Innov Technol Market Compl. 2021;7(3):173. doi: 10.3390/joitmc7030173

[42] Hassan A, Anwar I, Saleem A, Alalyani WR, Saleem I. Nexus between entrepreneurship education, motivations, and intention among Indian university students: the role of psychological and contextual factors. Indus Higher Edu. 2021;36(5):539–55. doi: 10.1177/09504222211053262

[43] Sweida G, Sherman CL. Does happiness launch more businesses? Affect, gender, and entrepreneurial intention. Int J Environ Res Public Health. 2020;17(18):6908. doi: 10.3390/ijerph17186908 32967308 PMC7559904

[44] Suehara VMB. Entrepreneurial abilities and attitude of business students as determinants of their interest in starting a business. Int J Eng Mgmt Res. 2023;13(1):12–34. doi: 10.31033/ijemr.13.1.3

[45] Huang Y, An L, Wang J, Chen Y, Wang S, Wang P. The role of entrepreneurship policy in college students’ entrepreneurial intention: the intermediary role of entrepreneurial practice and entrepreneurial spirit. Front Psychol. 2021;12:585698. doi: 10.3389/fpsyg.2021.585698 33776829 PMC7988310

[46] Požega Ž, Ribić D. The economic environment as a predictor of entrepreneurial intentions. Ekonomska misao i praksa. 2022;31(2):505–20. doi: 10.17818/emip/2022/2.8

[47] Wei Y. Regional governments and opportunity entrepreneurship in underdeveloped institutional environments: an entrepreneurial ecosystem perspective. Res Pol. 2022;51(1):104380. doi: 10.1016/j.respol.2021.104380

[48] Zhuang J, Sun H. Impact of institutional environment on entrepreneurial intention: the moderating role of entrepreneurship education. Inter J Manag Edu. 2023;21(3):100863. doi: 10.1016/j.ijme.2023.100863

[49] Neneh BN. Entrepreneurial passion and entrepreneurial intention: the role of social support and entrepreneurial self-efficacy. Stud Higher Edu. 2020;47(3):587–603. doi: 10.1080/03075079.2020.1770716

[50] Pinheiro GT, Moraes GHSM d, Fischer BB. Student entrepreneurship and perceptions on social norms and university environment: evidence from a developing country. J Entrep Emerg Econ. 2022;15(4):746–65.

[51] Yasir N, Mahmood N, Mehmood HS, Babar M, Irfan M, Liren A. Impact of environmental, social values and the consideration of future consequences for the development of a sustainable entrepreneurial intention. Sustainability. 2021;13(5):2648. doi: 10.3390/su13052648

[52] Pérez-Fernández H, Cacciotti G, Martín-Cruz N, Delgado-García JB. Are interactions between need for achievement and social networks the driving force behind entrepreneurial Intention? A trait activation story. J Business Res. 2022;149:65–76. doi: 10.1016/j.jbusres.2022.04.046

[53] Nisar S, Alshanberi AM, Mousa AH, El Said M, Hassan F, Rehman A, et al. Trend of social media use by undergraduate medical students; a comparison between medical students and educators. Ann Med Surg (Lond). 2022;81:104420. doi: 10.1016/j.amsu.2022.104420 36147096 PMC9486650

[54] Chen J, Liu L. Social media usage and entrepreneurial investment: an information-based view. J Busin Res. 2023;155:113423. doi: 10.1016/j.jbusres.2022.113423

[55] Loan NT, et al. Does social media foster students’ entrepreneurial intentions?. Cog Busin Manag. 2024;11(1):2298191.

[56] Al Halbusi H, Soto-Acosta P, Popa S. Analysing e-entrepreneurial intention from the theory of planned behaviour: the role of social media use and perceived social support. Int Entrep Manag J. 2023;19(4):1611–42. doi: 10.1007/s11365-023-00866-1

[57] Krueger NF, Reilly MD, Carsrud AL. Competing models of entrepreneurial intentions. J Busin Ventur. 2000;15(5–6):411–32.

[58] Barrera-Verdugo G, Villarroel-Villarroel A. Evaluating the relationship between social media use frequency and entrepreneurial perceptions and attitudes among students. Heliyon. 2022;8(4):e09214. doi: 10.1016/j.heliyon.2022.e09214 35434392 PMC9010631

[59] Lihua D. An Extended model of the theory of planned behavior: an empirical study of entrepreneurial intention and entrepreneurial behavior in college students. Front Psychol. 2022;12:627818. doi: 10.3389/fpsyg.2022.627818 35145443 PMC8823624

[60] Chen CC, Greene PG, Crick A. Does entrepreneurial self-efficacy distinguish entrepreneurs from managers?. J Business Ventur. 1998;13(4):295–316.

[61] Kurnia Khafidhatur R, Irsyad K, Matthew Olufemi A. The digital influence on entrepreneurial readiness: Exploring the role of social media and entrepreneurship education in enhancing self-efficacy. Indonesian J Business Entrep Res. 2025;3(1):27–37.

[62] Hu S, Jabor MK, Royo MA, Wu F. Social media use and entrepreneurial intention among vocational students in China: the role of entrepreneurial self-efficacy and gender. Int J Res Innov Soc Sci. 2024;VIII(XII):2689–709. doi: 10.47772/ijriss.2024.8120226

[63] Sousa-Filho JM de, Lessa B de S, Garcia-Salirrosas EE, Castro JL de C. The role of fear of failure on students’ entrepreneurial intentions in Latin America. Inter J Manag Edu. 2023;21(3):100880. doi: 10.1016/j.ijme.2023.100880

[64] Shaver KG, Scott LR. Person, process, choice: the psychology of new venture Creation. Entrep Theory Practice. 1992;16(2):23–46. doi: 10.1177/104225879201600204

[65] Cao Q. Entrepreneurial psychological quality and quality cultivation of college students in the higher education and moral education perspectives. Front Psychol. 2021;12:700334. doi: 10.3389/fpsyg.2021.700334 34539497 PMC8442845

[66] Sánchez LML, Plazas LAS, Ariza LR. The influence of emotional competencies on the entrepreneurship intentions of university students in Colombia. Sustainability. 2024;16(22):9933.

[67] Song Y, Lu G. The impact of university entrepreneurship support on college students’ entrepreneurial intention: a cognitive-affective perspective. Inter J Manag Edu. 2024;22(3):101087. doi: 10.1016/j.ijme.2024.101087

[68] Lim JY, Kim J, Kim S. The effects of the Start-Up NurseS program on nursing students using management strategy simulation. Nurse Educ Today. 2021;105:105020. doi: 10.1016/j.nedt.2021.105020 34217029

[69] Qudsia Yousaf H, Munawar S, Ahmed M, Rehman S. The effect of entrepreneurial education on entrepreneurial intention: the moderating role of culture. Inter J Manag Edu. 2022;20(3):100712. doi: 10.1016/j.ijme.2022.100712

[70] Pacheco JN, Turro A, Urbano D. Open social innovation: a systematic literature review and future research agenda. Technol Forecast Soc Change. 2025;216:124160. doi: 10.1016/j.techfore.2025.124160

[71] Huang Y, Zhang J. Social media use and entrepreneurial intention: the mediating role of self-efficacy. Soc Behav Pers. 2020;48(11):1–8. doi: 10.2224/sbp.9451

[72] Balgiu BA, Cotoară DM, Simionescu-Panait A. Validation of the Internet entrepreneurial self-efficacy scale among Romanian technical students. PLOS ONE. 2024;19(10):e0312929. 10.1371/journal.pone.0312929 PMC11527208 39480762

[73] Lim JY, Kim GM, Kim EJ. Predictors of entrepreneurial intention of nursing students based on theory of planned behavior. J Multidiscip Healthc. 2021;14:533–43. doi: 10.2147/JMDH.S288532 33664575 PMC7924248

[74] Sargani GR, Jiang Y, Zhou D, Chandio AA, Hussain M, Ali A, et al. How do gender disparities in entrepreneurial aspirations emerge in Pakistan? An approach to mediation and multi-group analysis. PLoS One. 2021;16(12):e0260437. doi: 10.1371/journal.pone.0260437 34874979 PMC8651106

[75] Wu H, Li S, Zheng J, Guo J. Medical students’ motivation and academic performance: the mediating roles of self-efficacy and learning engagement. Med Educ Online. 2020;25(1):1742964. doi: 10.1080/10872981.2020.1742964 32180537 PMC7144307

[76] Hair J, Hult GTM, Ringle CM, Sarstedt M. A primer on partial least squares structural equation modeling (PLS-SEM). 3rd ed. SAGE Publications, Inc.; 2021.

[77] Finstad K. Response interpolation and scale sensitivity: evidence against 5-point scales. J Usability Stud. 2010;5(3):104–10.

[78] Wakita T, Ueshima N, Noguchi H. Psychological distance between categories in the likert scale. Edu Psychol Measurem. 2012;72(4):533–46. doi: 10.1177/0013164411431162

[79] Zhang X, Abbas J, Shahzad MF, Shankar A, Ercisli S, Dobhal DC. Association between social media use and students’ academic performance through family bonding and collective learning: The moderating role of mental well-being. Educ Inf Technol. 2024;29(11):14059–89. doi: 10.1007/s10639-023-12407-y

[80] Tchokoté ID, Bawack R, Nana A. Attitude over norms: reevaluating the dominance of attitude in shaping entrepreneurial intentions among higher education students in Global South Countries. Inter J Manag Edu. 2025;23(2).

[81] Duong CD. A moderated mediation model of perceived barriers, entrepreneurial self-efficacy, intentions, and behaviors: a social cognitive career theory perspective. Oeconomia Copernicana. 2023;14(1):355–88. doi: 10.24136/oc.2023.010

[82] Schuberth F. The Henseler-Ogasawara specification of composites in structural equation modeling: a tutorial. Psychol Methods. 2023;28(4):843–59. doi: 10.1037/met0000432 34914475

[83] Mustafa YMH, Zami MS, Al-Amoudi OSB, Al-Osta MA, Wudil YS. Analysis of unconfined compressive strength of rammed earth mixes based on artificial neural network and statistical analysis. Materials (Basel). 2022;15(24):9029. doi: 10.3390/ma15249029 36556836 PMC9784941

[84] Movassagh AA, Alzubi JA, Gheisari M, Rahimi M, Mohan S, Abbasi AA, et al. Artificial neural networks training algorithm integrating invasive weed optimization with differential evolutionary model. J Ambient Intell Human Comput. 2021;14(5):6017–25. doi: 10.1007/s12652-020-02623-6

[85] Hair J, Hult T, Ringle C, Sarstedt M. A primer on partial least squares structural equation modeling (PLS-SEM). Thousand Oaks, CA: Sage Publications, Inc.; 2014.

[86] Goggins S, Xing W. Building models explaining student participation behavior in asynchronous online discussion. Comp Edu. 2016;94:241–51. doi: 10.1016/j.compedu.2015.11.002

[87] Urbach N, Ahlemann F. Structural equation modeling in information systems research using partial least squares. J Inform Technol Theory. 2010;11(2):2.

[88] Fornell C, Larcker DF. Evaluating structural equation models with unobservable variables and measurement error. J Market Res. 1981;18(1):39. doi: 10.2307/3151312

[89] Henseler J, Ringle CM, Sarstedt M. A new criterion for assessing discriminant validity in variance-based structural equation modeling. J Acad Mark Sci. 2014;43(1):115–35. doi: 10.1007/s11747-014-0403-8

[90] Nitzl C, Roldan JL, Cepeda G. Mediation analysis in partial least squares path modeling. Indus Manag Data Syst. 2016;116(9):1849–64. doi: 10.1108/imds-07-2015-0302

[91] Teo A-C, Tan GW-H, Ooi K-B, Hew T-S, Yew K-T. The effects of convenience and speed in m-payment. Indus Manag Data Syst. 2015;115(2):311–31. doi: 10.1108/imds-08-2014-0231

[92] Taneja A, Arora A. Modeling user preferences using neural networks and tensor factorization model. Inter J Inform Manag. 2019;45:132–48. doi: 10.1016/j.ijinfomgt.2018.10.010

[93] Sharma SK, Sharma H, Dwivedi YK. A hybrid SEM-neural network model for predicting determinants of mobile payment services. Inform Syst Manag. 2019;36(3):243–61. doi: 10.1080/10580530.2019.1620504

[94] EL Idrissi T, Idri A, Bakkoury Z. Systematic map and review of predictive techniques in diabetes self-management. Inter J Inform Manag. 2019;46:263–77. doi: 10.1016/j.ijinfomgt.2018.09.011

[95] Ooi KB, Tan GWH. Mobile technology acceptance model: an investigation using mobile users to explore smartphone credit card. Expert Syst Appl. 2016;59:33–46.

[96] Karaca Y, Moonis M, Zhang Y-D, Gezgez C. Mobile cloud computing based stroke healthcare system. Inter J Inform Manag. 2019;45:250–61. doi: 10.1016/j.ijinfomgt.2018.09.012

[97] Widjaja M, Dewi L. Pengaruh attitude toward behavioral, subjective norm, perceived behavioral control terhadap entrepreneurial intention. PERFORMA. 2023;8(1):10–9. doi: 10.37715/jp.v8i1.2018

[98] Batz Liñeiro A, Romero Ochoa JA, Montes de la Barrera J. Exploring entrepreneurial intentions and motivations: a comparative analysis of opportunity-driven and necessity-driven entrepreneurs. J Innov Entrep. 2024;13(1). doi: 10.1186/s13731-024-00366-8

[99] Wibowo A, Narmaditya BS, Suparno, Sebayang KDA, Mukhtar S, Shafiai MHM. How does digital entrepreneurship education promote entrepreneurial intention? The role of social media and entrepreneurial intuition. Soc Sci Human Open. 2023;8(1):100681. doi: 10.1016/j.ssaho.2023.100681

